# Biophysical Characterization of a Vaccine Candidate against HIV-1: The Transmembrane and Membrane Proximal Domains of HIV-1 gp41 as a Maltose Binding Protein Fusion

**DOI:** 10.1371/journal.pone.0136507

**Published:** 2015-08-21

**Authors:** Zhen Gong, Jose M. Martin-Garcia, Sasha M. Daskalova, Felicia M. Craciunescu, Lusheng Song, Katerina Dörner, Debra T. Hansen, Jay-How Yang, Joshua LaBaer, Brenda G. Hogue, Tsafrir S. Mor, Petra Fromme

**Affiliations:** 1 Department of Chemistry and Biochemistry, Arizona State University, Tempe, AZ 85287–1604, United States of America; 2 Center for Applied Structural Discovery, The Biodesign Institute, Arizona State University, Tempe, AZ 85287–1604, United States of America; 3 Center for Infectious Diseases and Vaccinology, The Biodesign Institute, Arizona State University, Tempe, Arizona 85287–5401, United States of America; 4 Center of Innovations in Medicine, The Biodesign Institute, Arizona State University, Tempe, Arizona 85287–5901, United States of America; 5 Virginia G. Piper Center for Personalized Diagnostics, The Biodesign Institute, Arizona State University, Tempe, AZ 85287–6401, United States of America; 6 School of Life Sciences, Arizona State University, Tempe, AZ 85287–4501, United States of America; University of Missouri-Kansas City, UNITED STATES

## Abstract

The membrane proximal region (MPR, residues 649–683) and transmembrane domain (TMD, residues 684–705) of the gp41 subunit of HIV-1’s envelope protein are highly conserved and are important in viral mucosal transmission, virus attachment and membrane fusion with target cells. Several structures of the trimeric membrane proximal external region (residues 662–683) of MPR have been reported at the atomic level; however, the atomic structure of the TMD still remains unknown. To elucidate the structure of both MPR and TMD, we expressed the region spanning both domains, MPR-TM (residues 649–705), in *Escherichia coli* as a fusion protein with maltose binding protein (MBP). MPR-TM was initially fused to the C-terminus of MBP via a 42 aa-long linker containing a TEV protease recognition site (MBP-linker-MPR-TM). Biophysical characterization indicated that the purified MBP-linker-MPR-TM protein was a monodisperse and stable candidate for crystallization. However, crystals of the MBP-linker-MPR-TM protein could not be obtained in extensive crystallization screens. It is possible that the 42 residue-long linker between MBP and MPR-TM was interfering with crystal formation. To test this hypothesis, the 42 residue-long linker was replaced with three alanine residues. The fusion protein, MBP-AAA-MPR-TM, was similarly purified and characterized. Significantly, both the MBP-linker-MPR-TM and MBP-AAA-MPR-TM proteins strongly interacted with broadly neutralizing monoclonal antibodies 2F5 and 4E10. With epitopes accessible to the broadly neutralizing antibodies, these MBP/MPR-TM recombinant proteins may be in immunologically relevant conformations that mimic a pre-hairpin intermediate of gp41.

## Introduction

The transmembrane (TM) domain of HIV-1 gp41 is one of the most highly conserved regions of the envelope glycoprotein (Env) of HIV-1 [1, 2]. This region is involved in many essential biological functions (recently reviewed by Steckbeck and co-workers [3]). The primary role of the gp41 TM domain is to anchor Env in both the viral and cellular membranes [4]. It has been recently reported that the TM domain also induces lipid mixing and associates with the fusion peptide of HIV-1 gp41 during the viral fusion process [5]. The gp41 TM peptide was able to inhibit virus-cell fusion because it associates strongly with the fusion peptide and thus may interfere with insertion of the fusion peptide into the target cell membrane, which makes the gp41 TM peptide a new and fascinating HIV-1 entry inhibitor [5]. Additionally, the gp41 TM domain shares a motif with the α subunit of the T-cell receptor TM domain [2]. The gp41 TM peptide co-localizes with CD3 in the T-cell receptor complex and inhibits T cell proliferation *in vitro*, and this interaction was suggested to be yet another strategy whereby HIV-1 evades immune responses [6].

Mao et al. reported a 6Å resolution cryo-electron microscopy structure of the membrane-bound HIV-1 envelope glycoprotein trimer in its uncleaved state, which included the TM domain of gp41 [7]. This report comprises the only structural information of the TM domain of gp41. The atomic structure of the gp41 TM domain remains unknown perhaps due to its high hydrophobicity making its expression, purification and crystallization difficult. The traditional model of the gp41 TM domain is a single membrane-spanning α-helix (residues 684–705) followed by an intracytoplasmic C-terminal tail [2]. An alternative model was proposed by Hollier and Dimmock [8] to explain the observation that an epitope in the C-terminal tail of HIV-1 gp41, the so-called Kennedy epitope [9], is extracellulary exposed under some conditions. To account for the drastic change in the membrane topology necessary for the exposure of the Kennedy epitope, Hollier and Dimmock suggested that under certain conditions the TM region assumes a different secondary structure consisting of three membrane-spanning β-sheets [8].

HIV-1 gp41 is essential in transcytosis, a process leading to mucosal transmission of the virus [10–12]. Transcytosis is initiated when gp41 binds to the epithelial glycosphingolipid galactosyl ceramide (GalCer), the epithelial cell receptor for HIV [13]. The minimal region required for gp41 to bind GalCer is the membrane proximal region (MPR, residues 650–685) [14]. MPR is another highly conserved segment of HIV-1 [2, 15] and contains the membrane proximal external region (MPER, residues 662–683) and part of the C-terminal heptad repeat region (CHR, residues 650–661). Furthermore, MPR is the target of secretory IgAs, which block HIV-1 transcytosis by neutralizing gp41 binding to GalCer [16, 17]. Significantly, epitopes in the MPER are recognized by three broadly neutralizing antibodies, 2F5 [18], 4E10 [19] and 10E8 [20].

HIV-1 gp41 mediates the membrane fusion between target cell and virus through its own conformational changes: from a native trimer prior to the interaction between gp120 and CD4, through a pre-hairpin intermediate, and then as a post-fusion trimer of hairpins (or a six-helix bundle) [21]. Structures of trimeric MPER have been solved in the pre-fusion [22] and post-fusion (six-helix bundle) conformations [23]. However, neither conformation could be recognized by the broadly neutralizing antibodies 2F5 or 4E10 [22, 23]. Another structure of trimeric MPER has been solved in the post-fusion state (a six-helix bundle) containing a shortened NHR (HR1) region, which leaves MPER accessible to the 2F5 antibody [24]. Recently, Reardon et al. reported an NMR structure of the trimeric MPER in a putative pre-fusion intermediate state (a three-helix bundle) [25]. In their structure, the N termini of the MPER helices are closely associated with each other while the C termini gradually separate, which leaves space for antibody binding. However, in their construct, MPER was fused to the C terminus of a 27-residue trimerization domain from bacteriophage T4 fibritin (the foldon domain). Although Reardon et al. reported that MPER was linked to the foldon motif through the flexible linker GSSG, which is intended to minimize the effect of the structured trimerization motif on the conformation and dynamics of MPER, it is still not experimentally confirmed that MPER forms a trimer in the pre-fusion intermediate form without the effect of the trimerization motif. Moreover, the tight association of the MPER trimer at the N terminus could be due to its proximity to the tight foldon motif while the C terminus of the MPER trimer separated from each other because the C terminus of the MPER trimer has less effect from the trimerization motif. To better understand HIV-1 gp41 and instruct design of vaccines and therapeutics against HIV-1, elucidating the structure of the gp41 MPR and TM domains is required.

Mistic, a *Bacillus subtilis* integral membrane protein [26], was previously used as a fusion partner in our laboratory to overexpress MPR-TM of HIV-1 gp41, upon which Mistic was removed for crystallization [27]. However, no crystals were obtained even after extensive crystallization screens. This result may be due to the highly hydrophobic property of MPR-TM and/or lack of crystal contacts between MPR-TM molecules. In the work reported here, a novel construct was designed to overexpress MPR-TM as a maltose binding protein (MBP) fusion. MBP is a commonly used fusion partner, capable of improving the solubility and expression level of the target protein [28–32]. MBP may function as a chaperone to assist the correct folding of the target protein in active form [28]. Furthermore, MBP may provide a large hydrophilic interaction surface for formation of crystal lattice contacts thereby facilitating crystal formation [33]. Structures of dozens of MBP recombinant proteins have been solved at atomic resolution by X-ray structure analysis in the past decades (reviewed in Refs. [33, 34]), which indicates that MBP could be used as a fusion partner for structural studies.

In the present study, we report the expression, purification and biophysical characterization of MPR-TM (residues 649 to 705) as a C-terminal fusion to the 8xHis-tagged MBP for structural determination by X-ray crystallography. Surface plasmon resonance (SPR) was used to test if the epitope on the purified protein was exposed and could be recognized by the broadly neutralizing mAbs 2F5 and 4E10. Crystals of the MBP/MPR-TM recombinant protein could not be obtained when MPR-TM was fused to the C-terminus of MBP via a 42-residue linker. The linker was changed to three alanine residues (MBP-AAA-MPR-TM) which may be more suitable for crystallization.

## Materials and Methods

### Cloning and expression of MBP-linker-MPR-TM and MBP-AAA-MPR-TM

The coding region of gp41 MPR-TM flanked by a tobacco etch virus (TEV) cleavage site was subcloned from pMistic-MPR-TM [27] into the pCR8/GW/Topo vector (Invitrogen, Carlsbad, CA), sequence-verified and shuttled into the pVP16 destination vector [35] by Gateway recombination cloning (Invitrogen, Carlsbad, CA). An aliquot of the LR reaction was used to transform One Shot TOP10 chemically competent E. coli cells following the protocol provided by the manufacturer (Invitrogen, Carlsbad, CA). Recombinant clones were selected on LB agar plates supplemented with 100 μg/mL of ampicillin. Plasmid DNA was isolated from several colonies using QIAGEN Plasmid Mini Kit (QIAGEN, Valencia, CA) and digested with *EcoR*I and *Kpn*I (Promega, Madison, WI) to confirm the presence of the insert in pVP16. Vectors and their sequences have been deposited and are available at the PSI:Biology-Materials Repository at DNASU (DNASU Plasmid ID: HiCD00674886) (http://dnasu.org) [36]. The verified recombinant expression vector of MBP-linker-MPR-TM was further transformed into NEB Express competent cells (New England Biolabs, Ipswich, MA), an enhanced BL21 derivative that allows inducible recombinant protein expression from T5 and other non-T7 promoters. Recombinant clones of MBP-linker-MPR-TM were selected on LB plates supplemented with 100 μg/mL of ampicillin.

To construct the gene encoding MBP-AAA-MPR-TM, a 153 bp *Bgl*II and *Sal*I restriction fragment that encompasses a region of the MBP-linker-MPR-TM coding sequence that encodes the long linker and a TEV protease recognition site (spanning EALKDAQ…ENLYFQG, S1 Fig) was replaced with a DNA sequence that encodes the protein sequence AALAAAQTNAAA. This cloning was accomplished with the following PCR reactions using Takara PrimeSTAR Max DNA Polymerase (Clontech, Mountain View, CA; see S1 Table for the primer sequences). PCR1 used primers MBP-MPR-fuseF1 and insert1-PCR1R, and template MBP-linker-MPR-TM; PCR2 used primers MBP-MPR-fuseF1 and insert1-PCR2R, and template PCR1; PCR3 used primers insert2-PCR1F and MBP-MPR-fuseR1, and template MBP-linker-MPR-TM; PCR4 used primers MBP-MPR-fuseF2 and MBP-MPR-fuseR1, and template PCR2. The products of PCR3 and PCR4 were simultaneously inserted into *Bgl*II- and *Sal*I-digested MBP-linker-MPR-TM using the ligation-independent InFusion HD Cloning Plus system (Clontech) [37]. DNA sequence confirmation was performed at the School of Life Sciences DNA Laboratory at Arizona State University. Vectors and their sequences have been deposited and are available from the PSI:Biology-Materials Repository at DNASU (DNASU Plasmid ID: HiCD00674843, http://dnasu.org) [36]. The verified recombinant expression vector of MBP-AAA-MPR-TM was further transformed into BL21(DE3) competent cells (Invitrogen). Recombinant clones of MBP-AAA-MPR-TM were selected on LB plates supplemented with 100 μg/mL of ampicillin.

Bacterial overexpression of both fusion proteins was done as follows. A single bacterial colony was used to inoculate a 100 mL pre-culture in LB containing 100 μg/mL of ampicillin in a shaker-incubator (37°C, 200 rpm) overnight. This culture was used to inoculate 3 L of LB broth containing 100 μg/mL of ampicillin at a dilution ratio of 1:30. Cells were grown at 37°C until the culture density reached OD_600_ between 0.6 and 0.8. Protein expression was induced by adding isopropyl β-D-1-thiogalactopyranoside (IPTG) to a final concentration of 500 μM and continued incubation for an additional 4 h. Cells were harvested by centrifugation (5000 *×g*, 10 min, 4°C) and cell pellets (12–14 g of wet cells per 3 L culture) were then stored at -80°C until further use.

### Preparation of the crude membrane fractions of MBP-linker-MPR-TM and MBP-AAA-MPR-TM

Preparation procedures of the crude membrane fractions of MBP-linker-MPR-TM and MBP-AAA-MPR-TM were the same. Cell pellets stored at -80°C were thawed and re-suspended in ice-cold phosphate buffered saline (PBS; 137 mM NaCl, 2.7 mM KCl, 10 mM Na2HPO4, 1.8 mM KH2PO4, pH 7.4) with 1× EDTA-free protease inhibitor (Sigma S8830). 100 mL of PBS buffer containing protease inhibitor was used to re-suspend 15 g of wet cell pellet. The suspension was disrupted in an ultrasonic homogenizer (Model 300 V/T, Biologics) at 150 W and 10 kHz for 1 min on and 1 min off while kept on ice. This step was repeated three times in total. The cell lysate was centrifuged at 20,000 *×g* for 20 min at 4°C. The supernatant was discarded and the pellet, containing the membrane fraction, peptidoglycan cell wall and other aqueous-insoluble material, was stored at -80°C overnight.

### Detergent solubilization of MBP-linker-MPR-TM and MBP-AAA-MPR-TM

The frozen crude membrane fraction of MBP-linker-MPR-TM was thawed and resuspended in ice-cold PBS buffer with protease inhibitor. 100 mL of PBS buffer containing protease inhibitor was used to re-suspend per 15 g of crude membrane fraction. Detergent screens were conducted by adding the following detergents to 1% (w/v) final concentration: lauryldimethylamine-oxide (LDAO), n-decyl-β-D-maltoside (β-DM), and n-dodecyl-β-D-maltoside (β-DDM). These suspensions were incubated at 4°C for 2 h. Screening for the optimal length of time required for efficient detergent extractions was done essentially as described above except that the incubation time was varied among 0.5 h, 1 h, 2 h and 3 h. Following their incubation, the suspensions were centrifuged at 20,000 *×g* for 20 min and the supernatant, which contained detergent-soluble proteins, was then used for further analyses. Large scale extractions were performed at using 1%βDDM at 4°C for 1 h.

### Purification of MBP-linker-MPR-TM and MBP-AAA-MPR-TM

Purification procedures of MBP-linker-MPR-TM and MBP-AAA-MPR-TM were the same. The detergent-solubilized proteins were purified by metal-affinity FPLC using a nickel-nitrilotriacetic acid (Ni-NTA) Superflow column (QIAGEN). The Ni-NTA Superflow resin was manually packed into the Tricon Empty 10/100 column (GE Healthcare, bed volume 8 mL). The column was equilibrated with buffer A (500 mM NaCl, 20 mM bicine, pH 8.0, and 0.05% β-DDM). The detergent soluble fraction was injected onto the Ni-NTA Superflow column by a 10 mL superloop (GE Healthcare) and washed with buffer A until the A_280_ was stable below 10 mAu. To remove non-specifically-bound proteins, the column was then washed with 2% buffer B (buffer A with 500 mM imidazole; final concentration of imidazole was 10 mM) until the A_280_ was stable below 20 mAu. Specifically-bound proteins were eluted from the column by application of a linear gradient of buffer B from 2% to 50% in 25 min at 1 mL/min. The eluted proteins were concentrated to 10–20 mg/mL using concentrators (100-kDa cutoff polyethersulfone membrane, Sartorius). Concentrated proteins were then subjected to size exclusion chromatography (SEC) using a Superdex-200 HR 10/300 gel filtration column (GE Healthcare) in SEC buffer (20 mM NaCl, 20 mM Tris-HCl, pH 7.5, 0.015% β-DDM). The Superdex-200 column was calibrated using the standards aprotinin (6.5 kDa), RNase A (13.7 kDa), carbonic anhydrase (29 kDa), ovalbumin (43 kDa), conalbumin (75 kDa), aldolase (158 kDa) and ferritin (440 kDa). Protein purity was analyzed by SDS-PAGE.

The protein concentration of MBP-linker-MPR-TM and MBP-AAA-MPR-TM were determined spectrophotometrically at 280 nm using molar extinction coefficients of 98,290 cm^-1^M^-1^ and 94,450 cm^-1^M^-1^, respectively.

### Cleaving MBP-linker-MPR-TM with TEV protease

After SEC purification, samples’ buffer conditions were adjusted to allow optimal TEV cleavage (final concentrations of 50 mM Tris-HCl, pH 8.0, 0.5 mM EDTA and 1 mM DTT). The recombinant protein MBP-linker-MPR-TM was digested with TEV protease (TEV: Substrate = 1 μg: 400 μg) [38] for 2 h at room temperature. Samples incubated in the same buffer without the TEV protease served as negative controls. The TEV protease cleavage products were subjected to a second Ni affinity purification step to purify the cleaved MPR-TM (collected in the flow-through, while the His-tagged MBP remains adsorbed to the column).

### SDS-PAGE and associated analyses

Proteins were resolved by SDS-PAGE and gels were either silver stained or subjected to immunoblotting as previously described [27]. Briefly, SDS-PAGE was performed with Tricine-SDS gels with 4% stacking and 8% separating gels. The primary antibody (1:2000 dilution) used in immunoblots was BSA-free monoclonal mouse Penta His antibody (QIAGEN), and the secondary antibody (1:2000 dilution) was goat anti-mouse polyclonal IgG-HRP (Invitrogen).

Purity of recombinant protein preparations were estimated by densitometric analysis of Coomassie-stained SDS-PAGE with ImageJ [39]. The gel was overloaded with 50 μg of MBP-linker-MPR-TM and MBP-AAA-MPR-TM proteins, which allowed more sensitive detection of contaminating proteins [40]. ImageJ was used to quantify the integrated density of each band and protein purity was calculated based on the density percentage.

### Clear Native PAGE analysis

NativePAGE^TM^ 4–16% Bis-Tris gel (Invitrogen) was used for clear native electrophoresis. Protein samples in the presence of 0.02% β-DDM were mixed with 2X native sample buffer (100 mM sodium chloride, 100 mM imidazole-HCl, 4 mM 6-aminohexanoic acid, 10% glycerol and 2 mM EDTA, pH 7.0) [41]. The cathode buffer contains 50 mM Tricine, 7.5 mM imidazole, pH 7.0 and the anode buffer contains 25 mM imidazole, pH 7.0 [42].

The relative mobilities (*R*_*f*_ *)* of protein standards were plotted against the known molecular mass (MW) values of the standards. Nonlinear regression was used to obtain the following formula: MW = 2054*e* ^-3.6Rf^ *-65* (R^2^ = 0.99); the formula in turn was used to interpolate the apparent molecular masses associated with the proteins bands.

The relative mobilities (*R*_*f*_ *)* of protein standards were plotted against the known molecular mass (MW) values of the standards. Nonlinear regression was used to obtain the following formula: MW = 2054*e* ^-3.6Rf^ *-65* (R^2^ = 0.99); the formula in turn was used to interpolate the apparent molecular masses associated with the proteins bands.

### Biophysical analyses

Dynamic light scattering (DLS) measurements were carried out using DynaPro NanoStar M3300 from Wyatt as previously described [27]. The sample’s polydispersity distribution is the standard deviation of the histogram that refers to the width of the peak [43]. The percent polydispersity (% polydispersity) is the polydispersity divided by the estimated hydrodynamic radius multiplied by 100. MALDI-TOF-MS was performed as previously described [27].

Circular dichroism (CD) spectra of purified proteins were measured by a JASCO J-710 CD spectropolarimeter [27]. Far UV CD spectra (185–260 nm) were recorded at 20°C in a 0.01 cm cuvette using the following parameters: scan speed, 50 nm/min; scanning increment, 0.5 nm; spectral bandwidth, 1.0 nm; response, 4 s. Reported spectra represent the average of 5 scans corrected for background solvent effects by subtraction of the appropriate blank and expressed as molar ellipticity. When comparing samples of MBP, MBP-Linker-MPR-TM and MPR-TM, proteins were diluted into 20 mM Tris-HCl, pH 7.5, 150 mM NaCl, and 0.02% β-DDM to an equimolar final concentration of 13 μM.

In order to study the effect of ionic strength on the secondary structure, the protein sample was concentrated to 5 mg/mL as described above and then diluted 20-fold into buffers containing 20 mM Tris-HCl, pH 7.5, 0.02% β-DDM and 0, 20, 50, 100, 150, 200, 250 or 300 mM NaCl. In order to identify the effect of pH on the secondary structure, the 5 mg/mL protein sample was diluted 20-fold into buffers containing 150 mM NaCl and 0.02% β-DDM at pH 6.5, 7.0, 7.5, 8.0 or 8.5. Prior to CD measurements, protein samples were incubated in the appropriate buffer overnight.

Analysis of the secondary structure content based on CD data was carried out using the CDPro software package [44]. Three methods CDSSTR [44–46], CONTIN/LL [47, 48], and SELCON3 [44, 49] were run using a protein reference set of 43 soluble proteins and 13 membrane proteins (SMP56) [44]. The average and standard deviation of the results from these three methods were calculated.

For thermal denaturation measurements monitored by CD spectroscopy, the CD signal at 220 nm was recorded from 15 to 90°C with a temperature change of slope at a rate of 2°C/min. The apparent midpoint temperature of the transition was calculated from the first derivative of the thermal melting curve.

### Surface plasmon resonance

**Fig 1:**
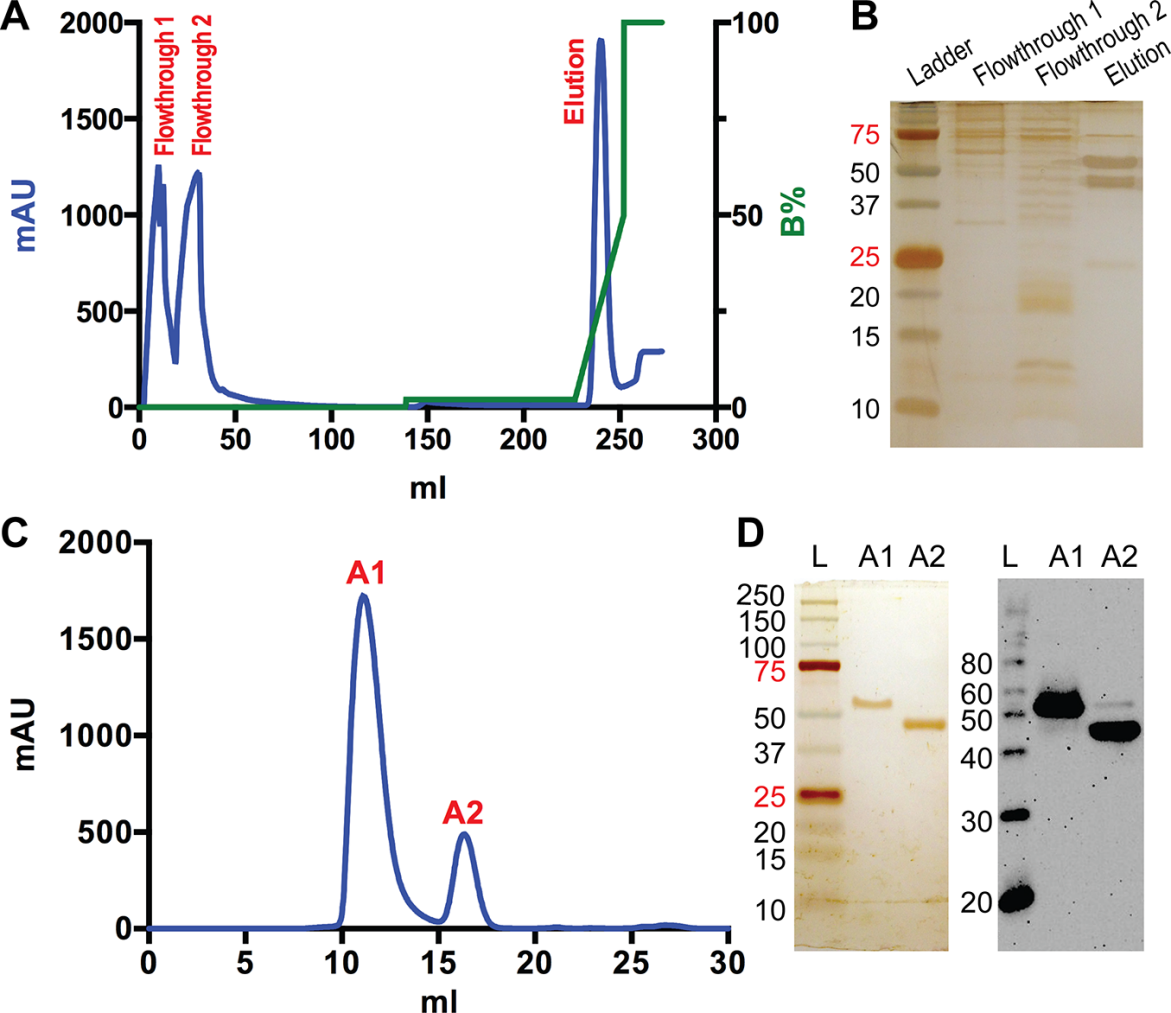
Purification of MBP-linker-MPR-TM. (A) Ni-affinity chromatogram of the βDDM extraction. Blue curve: UV absorbance at 280 nm; green curve: percentage of buffer B. (B) SDS-PAGE analysis of the flowthrough peaks and elution peak of the Ni-affinity chromatography. (C) SEC of the elution from Ni-affinity chromatography. Blue curve: UV absorbance at 280 nm. Peak A1 eluted at 11.1 mL and peak A2 eluted at 16.3 mL. (D) Silver stained SDS-PAGE (left) and anti-His Western blot (right) analysis of the peak A1 and A2 from SEC. L: molecular weight ladder.

All experiments were performed in quadruplicate on a KX5 Surface Plasmon Resonance Imaging (SPRi) System (Plexera) as previously described [27]. 10 μg/ml mAbs 2F5 (catalog number 1475) and 10 μg/ml 4E10 (catalog number 10091) were immobilized onto the surface of the gold chip (Plexera). The authors thank the NIH AIDS Research and Reference Reagent Program (Divisions of AIDS, NIAID, NIH) for donation of the mAbs 2F5 and 4E10. MBP (fractions A2 from SEC purification, Fig 1C) at 240 nM was used as a negative control. The purified MBP-linker-MPR-TM protein and MBP-AAA-MPR-TM protein were measured at 187 nM and 205 nM, respectively. Identical injections over blank surfaces were subtracted from the data for kinetic analyses.

## Results and Discussion

### Cloning and expression of MBP-linker-MPR-TM

In order to improve the expression level and solubility of the membrane proximal region and transmembrane (MPR-TM) domains of HIV-1 gp41, MPR-TM was fused to the C terminus of the maltose binding protein (MBP) by a 42 aa-long linker containing a tobacco etch virus (TEV) protease cleavage site (S1 Fig). An 8xHis-tag was cloned onto the N-terminus of MBP for purification purpose (S1 Fig). The MBP-linker-MPR-TM recombinant protein was expressed in NEB Express *E*. *coli*, a BL21 derivative-strain.

### Detergent solubilization of MBP-linker-MPR-TM

Anti-His immunoblot analyses of purification fractions indicated that most of the MBP-linker-MPR-TM fusion protein was in the water-insoluble fraction (S2A Fig). To establish the optimal solubilization conditions, we tested 1% LDAO, 1% β-DM and 1% β-DDM. The three detergents were able to extract the majority of the MBP-linker-MPR-TM protein from the pellet, and their extraction efficiencies were similar as judged by immunoblot analysis (S2B Fig). β-DDM was chosen for future purification because it has been widely and successfully used in crystallization of membrane proteins [50]. Analysis of the time needed for efficient detergent extraction showed that a 1 h incubation was sufficient to extract the majority of the MBP-linker-MPR-TM protein (S2C Fig).

### Purification of MBP-linker-MPR-TM

**Fig 2:**
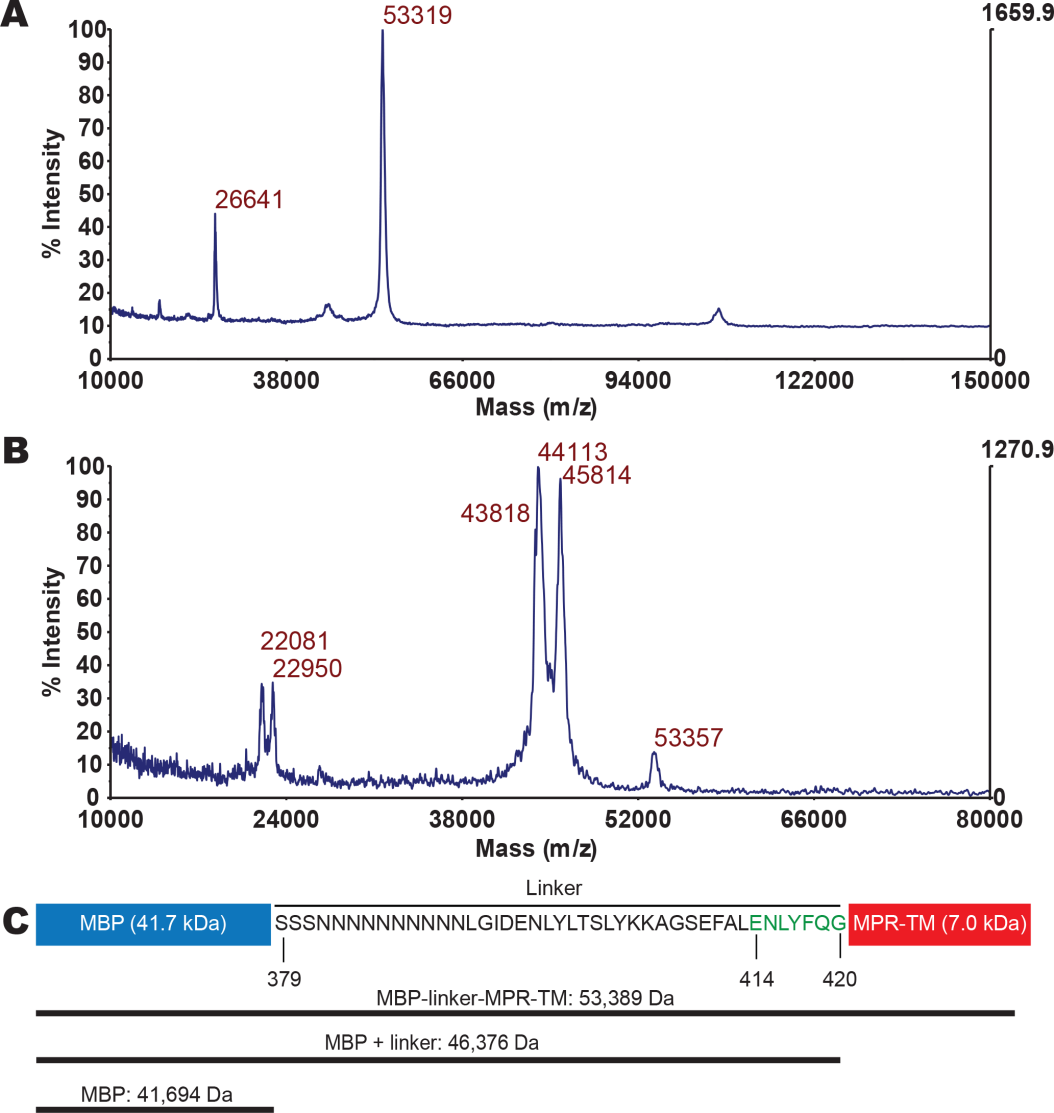
MALDI-TOF MS analysis of elutions from SEC. (A) MALDI-TOF MS analysis of the peak A1 from SEC (Fig 1C). (B) MALDI-TOF MS analysis of the peak A2 from SEC (Fig 1C). (C) Schematic representation of the MBP-linker-MPR-TM protein and predicted molecular weights of protein fragments. Residues 414–420 in green: TEV protease recognition site.

Purification of βDDM-solubilized MBP-linker-MPR-TM protein was accomplished by FPLC-connected Ni-affinity chromatography followed by size exclusion chromatography (SEC) in the presence of βDDM. A single elution peak was obtained from Ni-affinity column (Fig 1A). SDS-PAGE analysis of the flowthrough and elution peaks indicated two protein bands, one above the 50 kDa marker and one below (Fig 1B). These two proteins were successfully separated into peak A1 and peak A2 by SEC (Fig 1C) as determined by SDS-PAGE analysis (Fig 1D). Both bands were recognized by anti-His antibodies (Fig 1D), indicative of an intact 8xHis tag. MALDI-TOF MS analysis demonstrated that SEC peak A1 contains a protein of 53,319 Da (Fig 2A), possibly representing the MBP-linker-MPR-TM fusion protein (theoretical molecular weight: 53,389 Da). The signal at *m/z* 26,641 is likely to correspond to MBP-linker-MPR-TM charged with two protons. Peak A2 from the SEC purification contained proteins with masses ranging from 43,818 Da to 45,814 Da (Fig 2B). The theoretical molecular weight of the MBP itself is 41,694 Da (Fig 2C). MBP including the linker would have a molecular weight of 46,376 Da (Fig 2C). Therefore, proteins eluted in SEC peak A2 represent a number of C-terminally truncated versions of the recombinant fusion protein, which includes intact MBP (Fig 1C). The protein eluted in SEC peak A1 (Fig 1C) was used for further analyses. The purity of MBP-linker-MPR-TM, estimated by the densitometric analysis of an overloaded gel (S3A Fig), was >96%. The yield of MBP-linker-MPR-TM was approximately 60 mg per liter of culture.

### MBP-linker-MPR-TM forms oligomers and is monodisperse

SDS-PAGE and MALDI-TOF MS analysis can only be used to determine the molecular weight of the monomeric MBP-linker-MPR-TM because the proteins were denatured and lost their structural integrity and oligomeric state in these two analysis methods. The molecular weight of MBP-linker-MPR-TM in its detergent solubilized state was analyzed by analytical SEC, dynamic light scattering (DLS), and clear native PAGE.

The elution fraction from the Ni-affinity chromatography purification was analyzed by analytical SEC (Fig 1C). Based on the standard curve calibration, the A2 SEC peak (Fig 1C, centering at 16.3 mL) corresponded to proteins with MW of <50 kDa, which indicated that the various partially truncated fusion proteins containing intact MBP remained monomeric as has been previously reported for MBP [51]. The A1 SEC peak (Fig 1C, centering at 11.1 mL) corresponded to proteins with a MW of ~470 kDa based on our calibration. Considering that the proteins are embedded in large β-DDM micelles, the exact oligomeric state is difficult to assess, but is likely at least a hexamer. Previous studies demonstrated that MBP by itself is a monomer [51] but recombinant MBP fusion proteins may form oligomers depending on the nature of the fusion partner [29, 52, 53]. Our SEC results therefore provide an indication that it is the MPR-TM that is responsible for the oligomerization of MBP-linker-MPR-TM.

**Fig 3:**
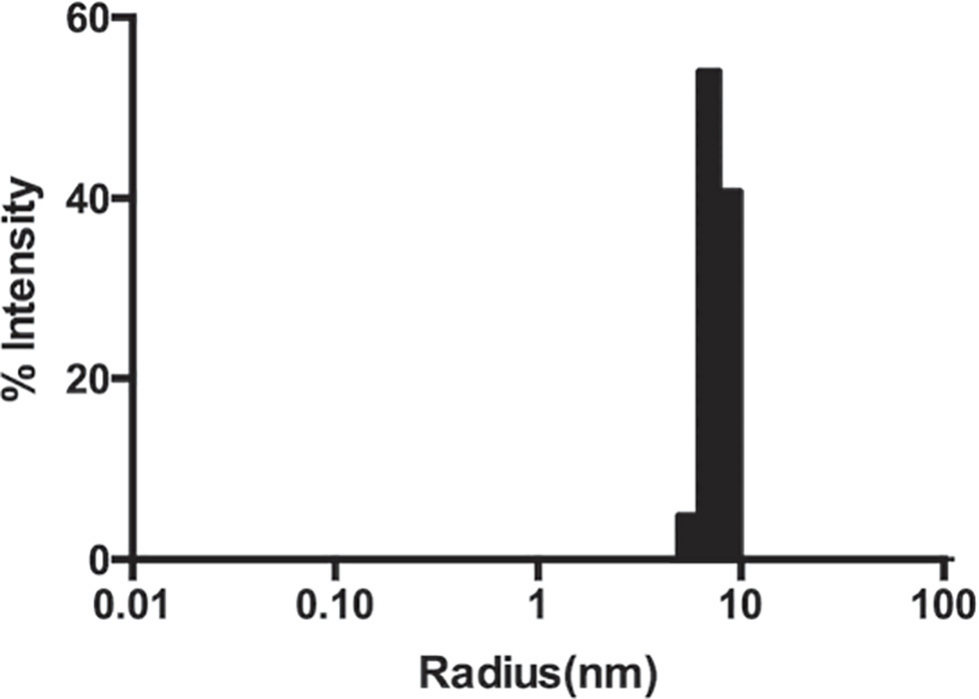
DLS measurement of 2 mg/ml MBP-linker-MPR-TM indicated monodispersity.

The size of a protein-detergent micelle estimated by SEC provides just a rough estimation of its size [54]. DLS was utilized in addition to estimate the molecular weight of MBP-linker-MPR-TM in the form of the protein-detergent complex (Fig 3). The hydrodynamic radius of the detergent-protein complex was 7.7 ± 0.5 nm, which corresponds to a molecular mass of about 400 kDa (S2 Table). This result is slightly different from the estimation by SEC (~470 kDa). Although both SEC and DLS can be used to roughly estimate the size of molecules in solution, the estimation by neither approach is accurate for membrane proteins because both DLS and SEC assume a spherical particle that is very likely not the case for our fusion protein. The most important function of DLS in our study is to measure the sample’s polydispersity distribution. The level of homogeneity is considered high when the percent polydispersity is less than 15% [55]. As shown in Fig 3 and S2 Table, the percent polydispersity of the peak is 13.4%, which indicated that the purified MBP-linker-MPR-TM is a monodisperse candidate for crystallization. It is concluded from the SEC and DLS analyses that MBP-linker-MPR-TM formed an oligomer in the presence of β-DDM. The presence of the detergent is vital for the stability of the protein in solution. This is evident when the fusion protein is subjected non-denaturing (native) PAGE analysis in the absence of detergents (S4 Fig). Under these conditions, the fusion protein, but not the MBP carrier protein by itself, is shown to maintain its oligomeric structure (S4 Fig, S4 Table). However, being partially stripped of its detergent layer, the oligomers tend to stack with regularity to form discrete complexes that resolve as a ladder (S4 Fig, S4 Table).

### Secondary structure estimation of MBP-linker-MPR-TM and MPR-TM by CD

**Fig 4:**
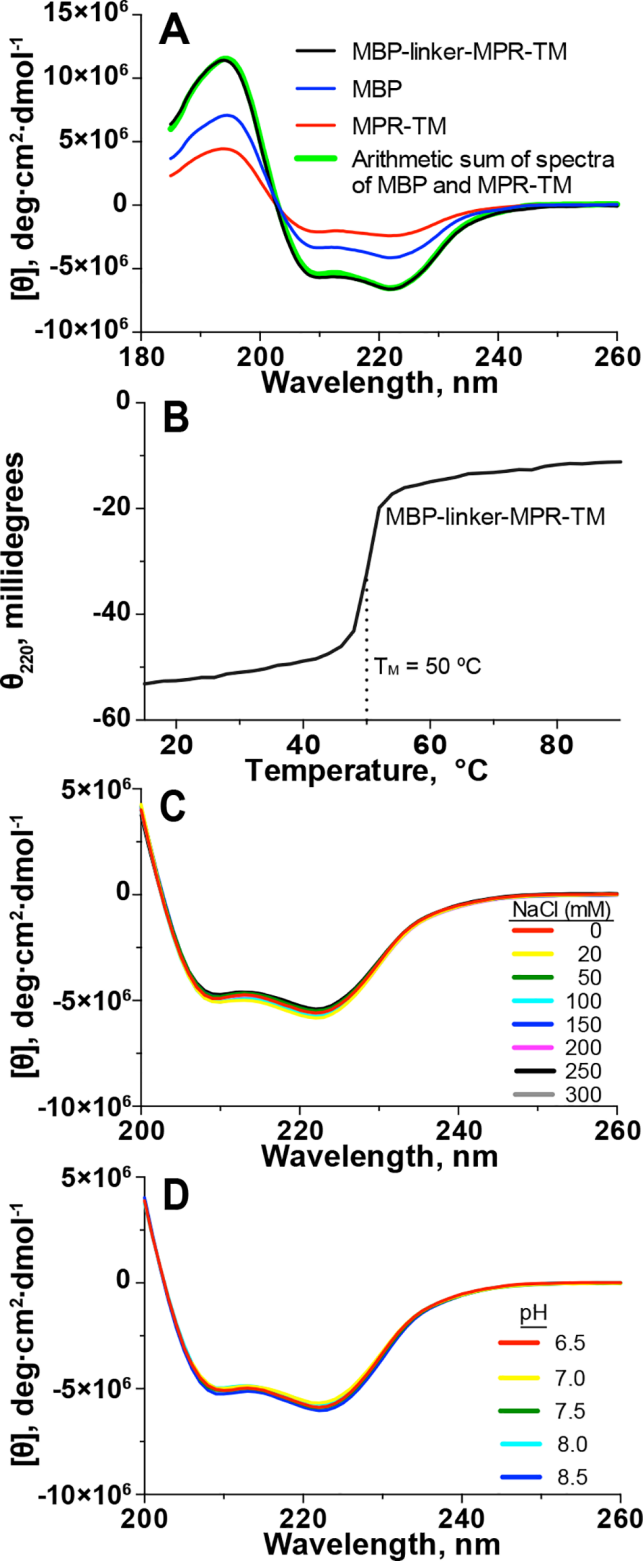
CD analysis of MBP-linker-MPR-TM, MPR-TM and MBP. (A) CD spectra of MBP-linker-MPR-TM, MPR-TM and MBP. Buffer: 100 mM NaF, 20 mM NaH_2_PO_4_, pH 7.5, and 0.02% βDDM. Protein concentration was 0.26 mg/mL. (B) Thermal denaturation curve of MBP-linker-MPR-TM measured at 220 nm and at a rate of temperature change of 2°C/min. The apparent midpoint “denaturation” temperature of the protein was 50°C. Protein concentration was 0.5 mg/mL. (C) Effect of ionic strength on the secondary structure of MBP-linker-MPR-TM. CD measurements were performed in 20 mM Tris, pH 7.5, 0.02% βDDM containing 0 to 300 mM NaCl. (D) Effect of pH on the secondary structure of MBP-linker-MPR-TM. CD spectra were recorded in 150 mM NaCl, 0.02% βDDM at pH 6.5 to 8.5.

It is essential to test if the purified MBP-linker-MPR-TM is folded well before carrying out crystallization screens. The results of CD measurement (Fig 4A, black trace) displayed one positive band at 193 nm and two negative bands at 208 and 222 nm, which is characteristic of a protein with a large fraction of α-helices [56]. Analysis of the secondary structure content of MBP-linker-MPR-TM by CDPro indicated the presence of 39.3 ± 2.3% α-helix, 13.5 ± 1.8% β-sheet and 47.3 ± 1.5% random coil. Comparison of the CD spectra of MBP (fraction A2 in Fig 1C) and MBP-linker-MPR-TM (fraction A1 in Fig 1C) demonstrated that the two minima of MBP-linker-MPR-TM were lower than that of MBP (Fig 4A, blue trace). Analysis of the secondary structure content of MBP by CDPro showed that MBP contained 33.0 ± 2.6% α-helix, 15.2 ± 2.4% β-sheet and 51.8 ± 1.2% random coil. The higher α-helix content in MBP-linker-MPR-TM might be due to the presence of MPR-TM.

To test the hypothesis that the HIV-1 MPR-TM domains contribute to the CD spectrum of the fusion protein, we separately measured the CD spectrum of MPR-TM that was cleaved from the fusion recombinant protein by TEV protease. The cleavage products contained cleaved MBP, MPR-TM and TEV protease (lane 2 in S5 Fig). Since the concentration of TEV protease used in the reaction was very low, the band of TEV protease could not be detected. A negative control without TEV protease was set up and analyzed by SDS-PAGE. In the negative control, MBP-linker-MPR-TM was not cleaved (lane 3 in S5 Fig). Subsequently, the TEV protease cleavage products were separated using a second Ni-affinity purification step. Cleaved MPR-TM was collected in the flow-though (lane 4 in S5 Fig) while the His-tagged MBP and TEV protease were retained on the column and subsequently eluted by imidazole (lane 5 in S5 Fig).

The CD spectrum of cleaved MPR-TM displayed one positive peak at 193 nm and two negative peaks at 208 and 222 nm (Fig 4A, red trace). Analysis of the secondary structure content of cleaved MPR-TM by CDPro indicated the presence of 76.3 ± 0.8% α-helix, 2.3 ± 1.0% β-sheet and 21.4 ± 1.7% random coil. The molar ellipticities of the MBP and MPR-TM (Fig 4A blue and red traces, respectively) are additive and the resultant curve is essentially identical with the measured curve of the fusion protein (Fig 4A green and black traces, respectively), suggesting the secondary structure of MPR-TM is not measurably affected within the context of the fusion protein. Moreover, our results fit published observations made with pre- and post-fusion Env using crystallography and cryo-electron microscopy [7, 22–24].

### Thermal stability of MBP-linker-MPR-TM

In order to estimate the thermal stability of the MBP-linker-MPR-TM protein and to guide decisions of the optimal temperature at which crystallization screens should be performed, CD spectroscopy was used. The CD spectra showed that the apparent midpoint “denaturation” temperature (T_M_) of the sample was 50°C (Fig 4B), which indicated good thermal stability. According to the melting curve obtained, the MBP-linker-MPR-TM protein would not denature below 45°C (Fig 4B). Therefore, crystallization screens of MBP-linker-MPR-TM could be performed at room temperature or lower temperatures.

### Effect of ionic strength and pH on the secondary structure of MBP-linker-MPR-TM

Ionic strength and pH are two important parameters which could be adjusted in crystallization screens to promote crystal formation. We used CD measurements to investigate the effect of ionic strength and pH on the secondary structure of the MBP-linker-MPR-TM protein to determine the range of these two parameters in which the protein would be correctly folded under crystallization conditions. The purified MBP-linker-MPR-TM protein was concentrated to 5 mg/mL and diluted into buffers containing 0 to 300 mM NaCl at pH 7.5 (Fig 4C) or diluted into buffers containing 150 mM NaCl at pH values ranging from 6.5 to 8.5 (Fig 4D).

The CD spectra of MBP-linker-MPR-TM in buffers containing different ionic strength (Fig 4C) and pH (Fig 4D) all showed negative bands at 208 and 222 nm of almost the same molar ellipticity. Our results indicate that the fusion protein MBP-linker-MPR-TM is stable under all tested conditions. Therefore, crystallization screens of MBP-linker-MPR-TM could potentially be carried out at a large range of ionic strength and pH, at which the protein will not be denatured.

### MBP-linker-MPR-TM is stable at high protein concentration

Crystallization screens often start with a protein concentration at 10 mg/mL, although the optimal concentration for each protein should be experimentally determined as it depends on many factors such as molecular weight and the stability of the protein at high concentration. Here, we used SEC and DLS to test the stability of MBP-linker-MPR-TM at high protein concentration. The protein was concentrated to 10 mg/mL by a 100-kDa cut-off concentrator to avoid concentration of the detergent βDDM, whose micelle molecular weight is about 70 kDa [27]. The concentrated sample was stored at 4°C and analyzed by SEC and DLS at day 1, 3 and 7.

**Fig 5:**
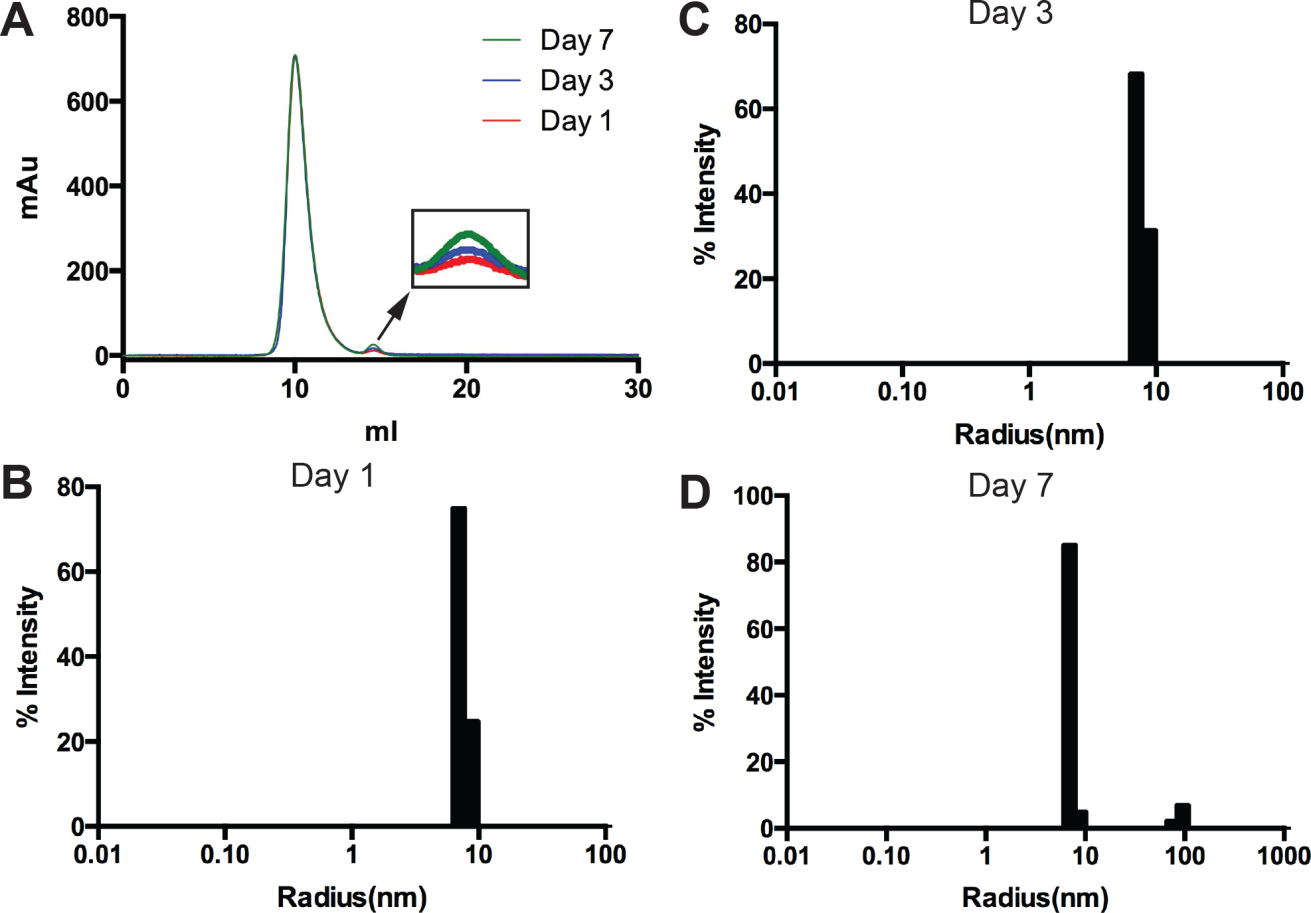
Stability test of 10 mg/mL MBP-linker-MPR-TM. MBP-linker-MPR-TM (10 mg/mL) was stored at 4°C and measured by SEC and DLS on day 1, 3 and 7. (A) Stability test of 10 mg/mL MBP-linker-MPR-TM by SEC. Insert: magnification of the degradation peak, which indicated slight increase of the MBP-linker-MPR-TM degradation from day 1 to day 7. (B-D) Stability test of 10 mg/mL MBP-linker-MPR-TM by DLS. The protein sample was homogeneous and monodisperse for 7 days at 4°C; only a little protein aggregation was detected on day 7.

The size exclusion chromatogram (Fig 5A) revealed a main peak eluting at about 10 mL and a very small minor peak eluting at 14.5 mL from day 1 to day 7. The main peak was expected to contain oligomeric MBP-linker-MPR-TM/detergent complex while the small minor peak at 14.5 mL might represent the MBP protein. The small minor peak increased slightly from day 1 to day 7, which represented 0.4%, 0.7% and 1.5% of the total area respectively (insert in Fig 5A), but was still very small compared with the main peak after one week storage at 4°C.

The DLS measurements (Fig 5B, 5C and 5D and S4 Table) revealed a narrow peak at 7.1 to 7.6 nm with the percent polydispersity below 12% from day 1 to day 7, which indicates that the protein sample is in a monodisperse condition for seven days at 4°C (Fig 5B, 5C and 5D and S4 Table). Although a small aggregation peak at 90.2 nm was detected on day 7, the aggregation peak represented only 0.1% of the total amount (mass) of the protein because the increase in scattered intensity is proportional to r^6^ (r is the particle radius) [57]. In conclusion, at a concentration of 10 mg/mL, MBP-linker-MPR-TM was homogeneous and monodisperse for 7 days, and therefore crystallization screens could be performed at this concentration.

### MBP-linker-MPR-TM is recognized by the broadly neutralizing mAbs 2F5 and 4E10

An important feature of gp41 is that it contains the epitopes for broadly neutralizing antibodies 2F5 and 4E10, which makes gp41 an attractive target for vaccine design. However, the epitopes for 2F5 and 4E10 cannot bind the antibodies in the pre-fusion and post-fusion conformations of gp41 [22, 23]. HIV-1 gp41 mediates the membrane fusion between target cell and virus through its own conformational change: native trimer prior to the interaction between gp120 and CD4, pre-hairpin intermediate and post-fusion trimer of hairpins (or a six-helix bundle) [21]. It was reported by Frey et al that gp41 in its prefusion conformation could not interact with 2F5 or 4E10: gp41 in its post-fusion conformation binds 2F5 very weakly (*K*_*D*_ ≈ 1.4 μM), while gp41 in its pre-hairpin intermediate state binds 2F5 and 4E10 very strongly (*K*_*D*_ < 10 nM) [58]. Therefore, it is of great interest to measure the binding affinities of MBP-linker-MPR-TM to 2F5 and 4E10 by surface plasmon resonance (SPR), from which the conformational information of MPR-TM could be estimated.

**Fig 6:**
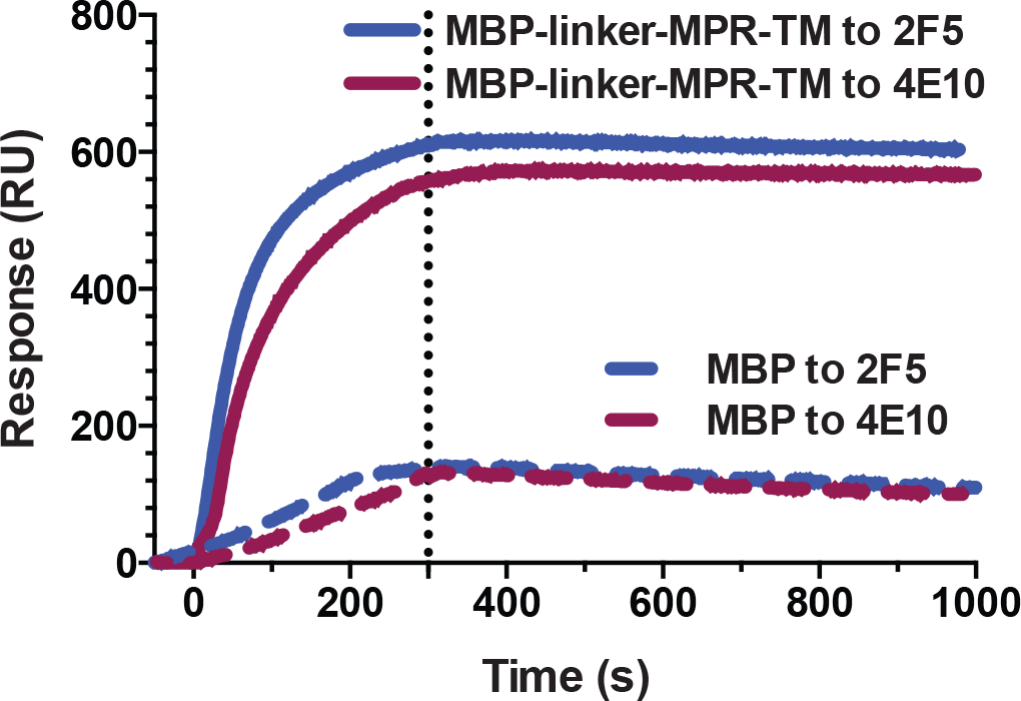
Surface plasmon resonance analysis of MBP-linker-MPR-TM. The mAbs 2F5 and 4E10 were immobilized onto the surface of a gold chip (Plexera) and the purified MBP-linker-MPR-TM protein was the analyte. MBP (fractions A2 from SEC purification, Fig 1C) was used as a negative control.

SPR measurements showed that mAbs 2F5 and 4E10 bound strongly to MBP-linker-MPR-TM with a *K*_*D*_ of 0.5 nM for the 2F5 Ab and a *K*_*D*_ of 0.4 nM for the 4E10 Ab (Fig 6 and Table 1). This result suggests that MBP-linker-MPR-TM may be in the pre-hairpin intermediate conformation, at which stage the epitopes for 2F5 and 4E10 are exposed and are available for antibody binding. The weak unspecific binding of 2F5 and 4E10 to the negative control MBP (fractions A2 from SEC purification, Fig 1C) may be due to the existence of high concentration of maltose (10% maltose) in the antibody solution. However, the binding affinities of MBP-linker-MPR-TM to mAbs 2F5 and 4E10 are much stronger than that of MBP. Therefore, the strong binding of MBP-linker-MPR-TM to mAbs 2F5 and 4E10 is due to the presence of MPR-TM instead of MBP.

**Table 1:** Binding rate constants of MBP-linker-MPR-TM derived from SPR analysis^a^.

Immobilized ligand	Flowing analyte	*k*_a_, M^-1^ s^-1^ (10^4^)	*k*_*d*_, s^-1^ (10^−5^)	*K*_*D*_, nM
2F5	MBP-linker-MPR-TM	7.0 ± 1.2	3.5 ± 1.2	0.5 ± 0.2
4E10	MBP-linker-MPR-TM	4.8 ± 0.1	1.8 ± 0.5	0.4 ± 0.1

^a^Results are the average of four independent measurements and are listed as mean ± SD.

### Alternation of the linker of MBP-linker-MPR-TM to three alanine residues

**Fig 7:**
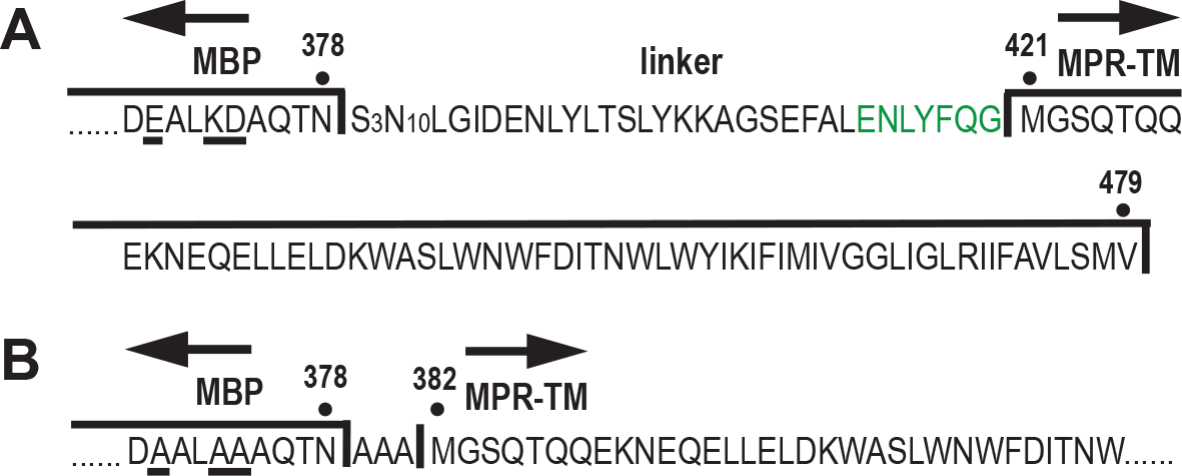
Schematic representation of the sequence of MBP-linker-MPR-TM (A) and MBP-AAA-MPR-TM (B). Amino acid changes in the C-terminus of MBP are underlined. Green: TEV protease recognition site (residues 414–420).

Extensive crystallization trials with screens of thousands of conditions using both vapor diffusion method and liquid cubic phase did not yield crystals of MBP-linker-MPR-TM with diffraction quality. Similar problems were reported by Center et al., who described that crystals could not be obtained when the ectodomain of gp21 from human T cell leukemia virus type I upon its fusion to the C-terminus of MBP via a flexible linker containing 25 residues until the linker was changed to three alanine residues [59]. We applied their strategy to our study. Therefore, the 42-residue-long linker containing a TEV protease recognition site was replaced by a short, three-alanine linker (Fig 7). In addition, three charged residues at the C-terminus of MBP (Glu-370, Lys-373 and Asp-374) were replaced by alanine residues to avoid potential electrostatic repulsion between MBP monomers in the event that MPR-TM forms a trimer [59]. The new fusion protein was dubbed MBP-AAA-MPR-TM.

**Fig 8:**
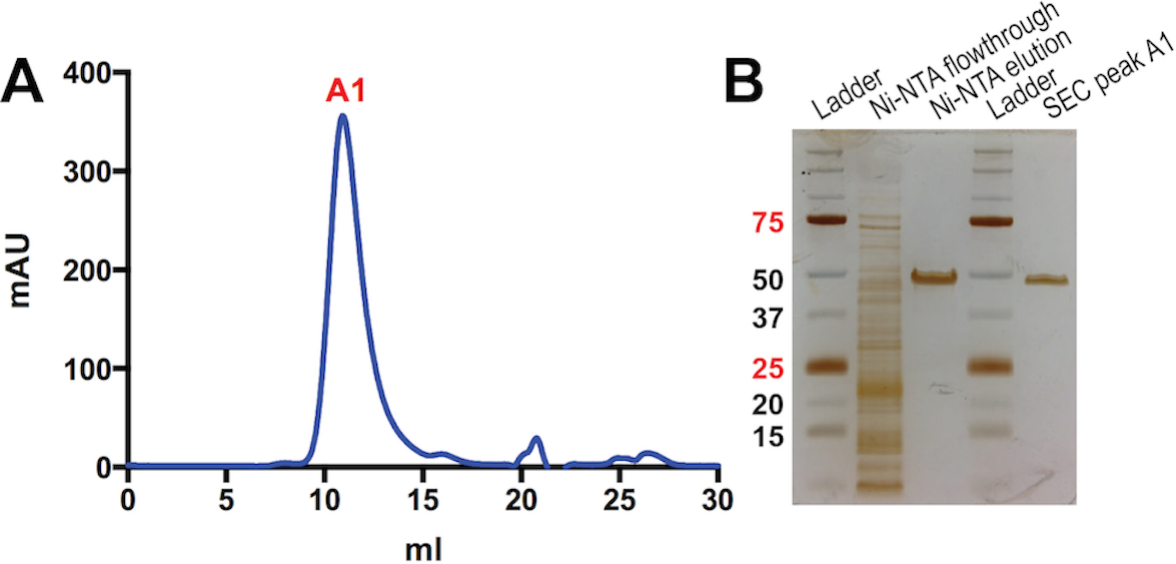
Purification of MBP-AAA-MPR-TM. (A) SEC of the Ni-affinity elution of MBP-AAA-MPR-TM. (B) Silver stained SDS-PAGE analysis of purification fractions.

We employed the purification procedure that was devised for MBP-linker-MPR-TM to purify MBP-AAA-MPR-TM. Ni-affinity chromatography of MBP-AAA-MPR-TM (Fig 8A) resulted in a single band at the expected apparent MW (49 kDa, Fig 8B), in contrast to preparations of the longer-linker fusion protein, which contained prominent contaminating degradation products (Fig 1A and 1B). The purity of the affinity chromatography eluate was further demonstrated by a subsequent SEC, which exhibited a single peak (Fig 8A). The purity of MBP-AAA-MPR-TM was almost 100%, estimated by the densitometry (S3B Fig). About 60 mg of pure MBP-AAA-MPR-TM protein was obtained from 1 liter of bacterial culture.

### CD, DLS and SPR measurements of MBP-AAA-MPR-TM

**Fig 9:**
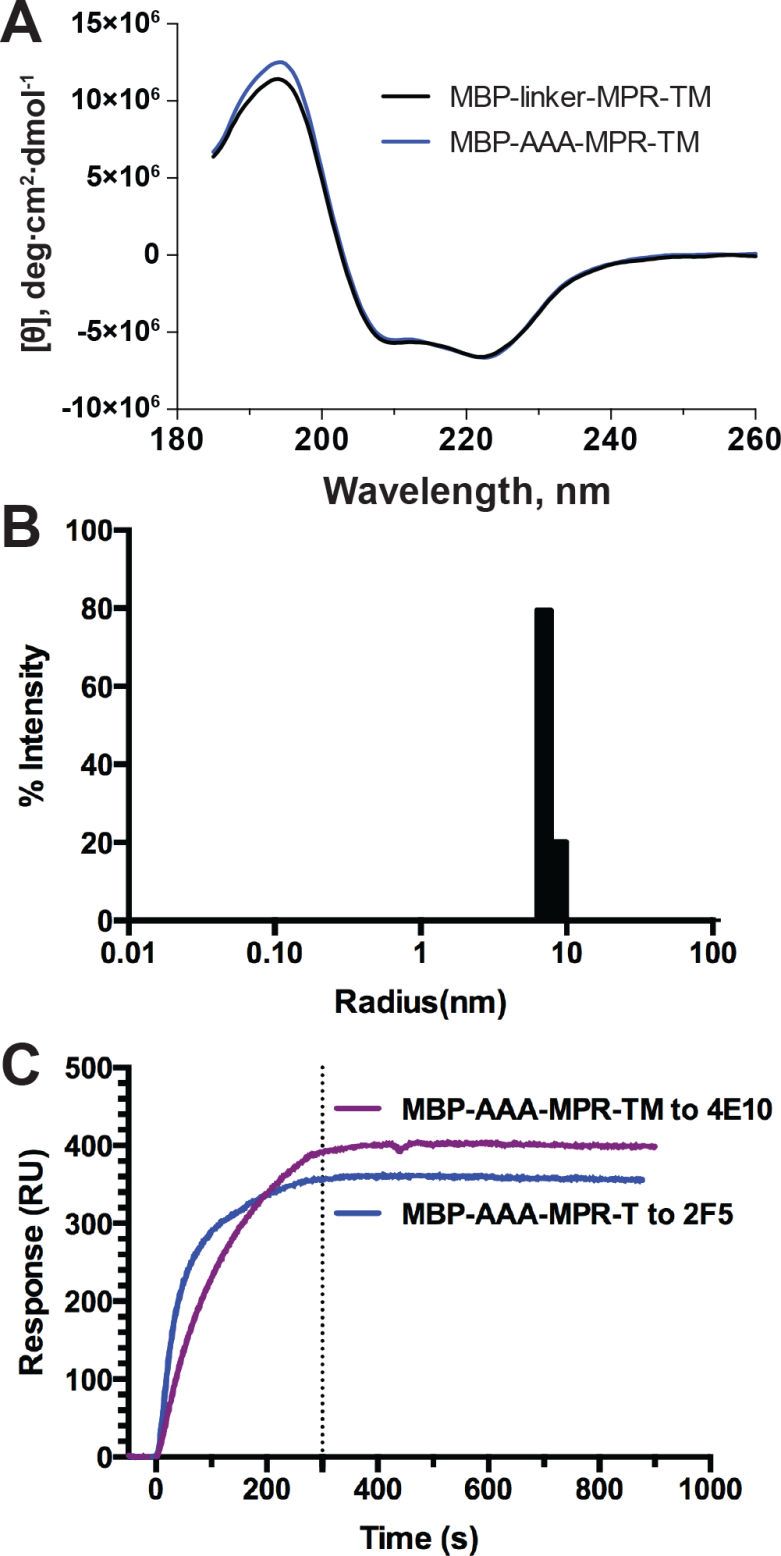
CD, DLS and SPR measurements of MBP-AAA-MPR-TM. (A) Comparison of CD spectra of MBP-linker-MPR-TM and MBP-AAA-MPR-TM. (B) DLS measurement of 1 mg/ml MBP-AAA-MPR-TM showed one monodisperse peak at 7.4 ± 0.8 nm. (C) SPR analysis. The mAbs 2F5 and 4E10 were immobilized onto the surface of a gold chip (Plexera) and the purified MBP-AAA-MPR-TM protein was the analyte.

CD spectroscopy measurements showed that the CD spectrum of MBP-AAA-MPR-TM was very similar to that of MBP-linker-MPR-TM (Fig 9A). Analysis of the secondary structure content of MBP-AAA-MPR-TM by CDPro indicated 38.8 ± 2.3% α-helix, 13.9 ± 2.6% β-sheet and 47.4 ± 2.6% random coil, which is in good agreement with the secondary structure content of MBP-linker-MPR-TM (39.3 ± 2.3% α-helix, 13.5 ± 1.8% β-sheet and 47.3 ± 1.5% random coil). These results indicate that changing of the linker to three alanine residues did not affect the secondary structure of the recombinant protein.

DLS analysis displayed one monomeric peak at 7.4 ± 0.4 nm, which corresponded to a protein/detergent complex of 360 kDa (Fig 9B and S5 Table). The size estimation of MBP-AAA-MPR-TM is slightly smaller than that of MBP-linker-MPR-TM (7.7 ± 0.5 nm and ~400 kDa, Fig 3 and S2 Table), which reflects the slightly smaller mass of MBP-AAA-MPR-TM.

As the longer linker could provide more flexibility of the MPR region for binding the broadly neutralizing antibodies, we conducted SPR measurements to determine if the MBP-AAA-MPR-TM protein still binds the antibodies with high affinity. The SPR result shown in Table 2 indicated that both 2F5 and 4E10 bind to MBP-AAA-MPR-TM with nanomolar to subnanomolar affinities: *K*_*D*_ values of 1.0 nM and 0.5 nM were determined for 2F5 and 4E10, respectively (Fig 9C and Table 2). It is therefore concluded that MBP-AAA-MPR-TM protein binds 2F5 and 4E10 antibodies with nanomolar to sub-nanomolar affinities, which suggests it may be suitable as a component in a future vaccine against HIV-1.

**Table 2:** Binding rate constants of MBP-AAA-MPR-TM derived from SPR analysis^a^.

Immobilized ligand	Flowing analyte	*k*_a_, M^-1^ s^-1^ (10^4^)		*k*_*d*_, s^-1^ (10^−5^)	*K*_*D*_, nM
2F5	MBP-AAA-MPR-TM	2.1 ± 0.3	2.1 ± 0.8		1.0 ± 0.5
4E10	MBP-AAA-MPR-TM	0.73 ± 0.13	0.37 ± 0.18		0.5 ± 0.1

^a^Results are the average of four independent measurements and are listed as mean ± SD.

### Conclusion

In summary, we describe here the expression and purification of two recombinant protein variants consisting of a fusion between MBP and MPR-TM of HIV-1 gp41. In one of these variants, MPR-TM was fused to the C-terminus of MBP via a 42-aa-long linker containing a TEV protease recognition site. In the second variant, the long linker was replaced by a short and structured peptide consisting of three alanine residues. Both proteins were purified to homogeneity and were shown to be stable in solution under various conditions and to remain monodisperse over time. Both proteins were able to strongly interact in solution with the broadly neutralizing mAbs 2F5 and 4E10 with nanomolar to sub-nanomolar affinities, in good agreement with our previously published results concerning MPR-TM [27]. The longer linker variant MBP-linker-MPR-TM was not amenable to crystallization under exhaustive screening. Crystallization experiments of MBP-AAA-MPR-TM are currently underway.

## Supporting Information

S1 Fig Amino acid sequence of MBP-linker-MPR-TM. The color scheme is as follows: blue, 8His-MBP (residues 1–378); black and green: 42 aa-long linker (residues 379–420) containing a TEV protease recognition site; green: TEV protease recognition site (residues 414–420); red: MPR-TM (residues 421–479).
(EPS)

S2 Fig Anti-His Western blot analysis of the detergent solublization of MBP-linker-MPR-TM. (A) Cell lysate fractions. (B) Comparison of the extraction efficiency of different detergents. (C) Determination of the time needed for efficient detergent extraction.
(TIF)

S3 Fig Purity estimation of MBP-linker-MPR-TM and MBP-AAA-MPR-TM. SDS-PAGE was overloaded with 50 μg of MBP-linker-MPR-TM and MBP-AAA-MPR-TM proteins for a more sensitive detection of protein impurities.
(TIF)

S4 Fig Clear native PAGE analysis of MBP-linker-MPR-TM and MBP-AAA-MPR-TM. Native gels were prepared as described under Materials and Methods. Considering the very low polydispersity of our preparations seen by both SEC and DLS, it was expected that subjecting the protein to electrophoresis under non-denaturing conditions would result in a single band corresponding to the oligomeric protein. However, when preparations of the fusion protein were subjected to clear native PAGE (S4 Fig) as well as other nondenaturing PAGE protocols (data not shown) [41, 42] we observed a ladder pattern indicating multiple oligomeric forms. In contrast, the cleaved MBP fusion partner resolves as a monomer (S4 Fig). Pre-stained protein standards resolve according to their molecular masses, however the MBP-linker-MPR-TM protein migrate according to a more complex (largely empiric) function of its charge and mass. In addition, the electrophoresis was conducted in the absence of detergents (in either the gel or the running buffer), and the β-DDM present in the protein samples was expected to be progressively diluted during the run. Consequently, assessing the molecular mass of the protein bands and determining their oligomeric configuration is speculative and is probably a consequence of the PAGE. Two plausible interpretations are offered in S3 Table.
(TIF)

S5 Fig Generation by cleavage and purification of MPR-TM. SDS-PAGE analysis of purification fractions stained by coomassie blue. Lane 1: proteins standards; lane 2: TEV cleavage products; lane 3: negative control (no TEV protease was added); lane 4: Ni-NTA flowthrough containing MPR-TM; lane 5: Ni-NTA elution containing MBP and TEV protease.
(TIF)

S1 Table Primer sequences. (DOCX)

S2 Table DLS measurement of purified MBP-linker-MPR-TM. (DOCX)

S3 Table MPR-TM fusion proteins resolve as regularly spaced bands by clear native PAGE. (DOCX)

S4 Table DLS measurements of MBP-linker-MPR-TM (10 mg/mL) subjected to prolonged incubation at 4°C. (DOCX)

S5 Table DLS measurement of purified MBP-AAA-MPR-TM. (DOCX)
