# Supplementary figures and images for: Biophysical Characterization of a Vaccine Candidate against HIV-1: The Transmembrane and Membrane Proximal Domains of HIV-1 gp41 as a Maltose Binding Protein Fusion
Source: PLoS One. 2015 Aug 21;10(8):e0136507. doi: 10.1371/journal.pone.0136507 (PMC4546420; doi:10.1371/journal.pone.0136507)

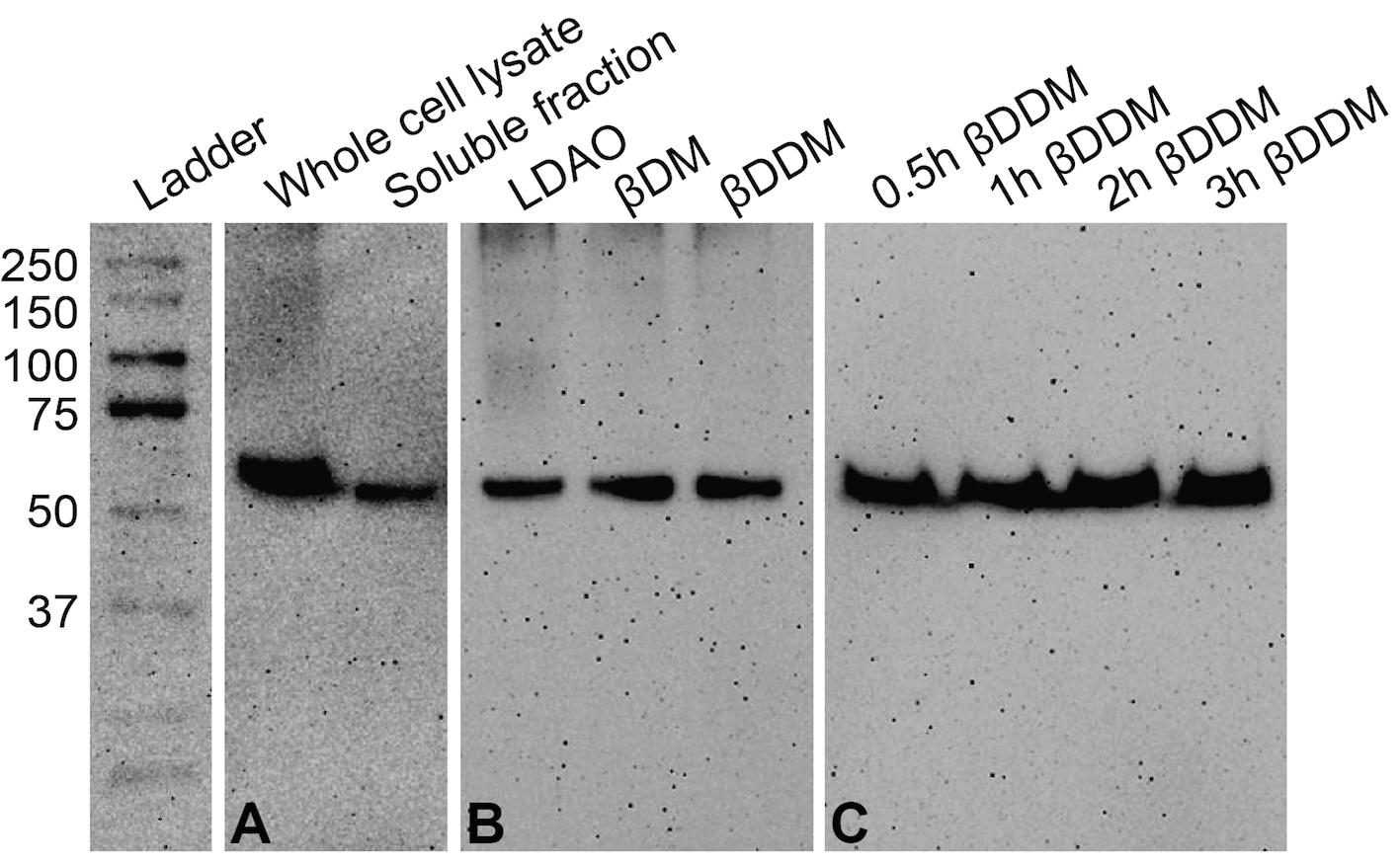

Supplement: S2 Fig — (A) Cell lysate fractions. (B) Comparison of the extraction efficiency of different detergents. (C) Determination of the time needed for efficient detergent extraction. (TIF) [file pone.0136507.s002.tif]

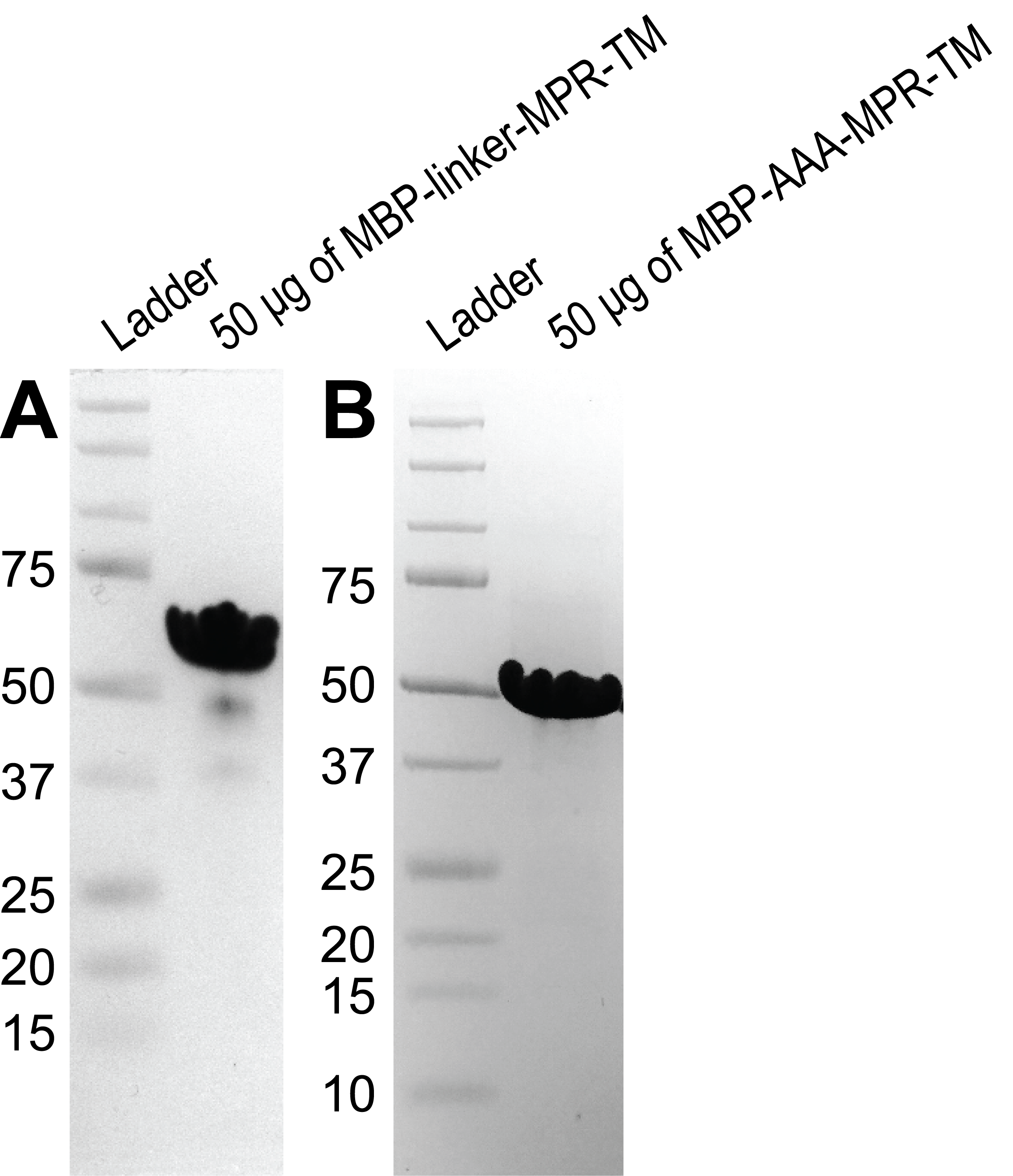

Supplement: S3 Fig — SDS-PAGE was overloaded with 50 μg of MBP-linker-MPR-TM and MBP-AAA-MPR-TM proteins for a more sensitive detection of protein impurities. (TIF) [file pone.0136507.s003.tif]

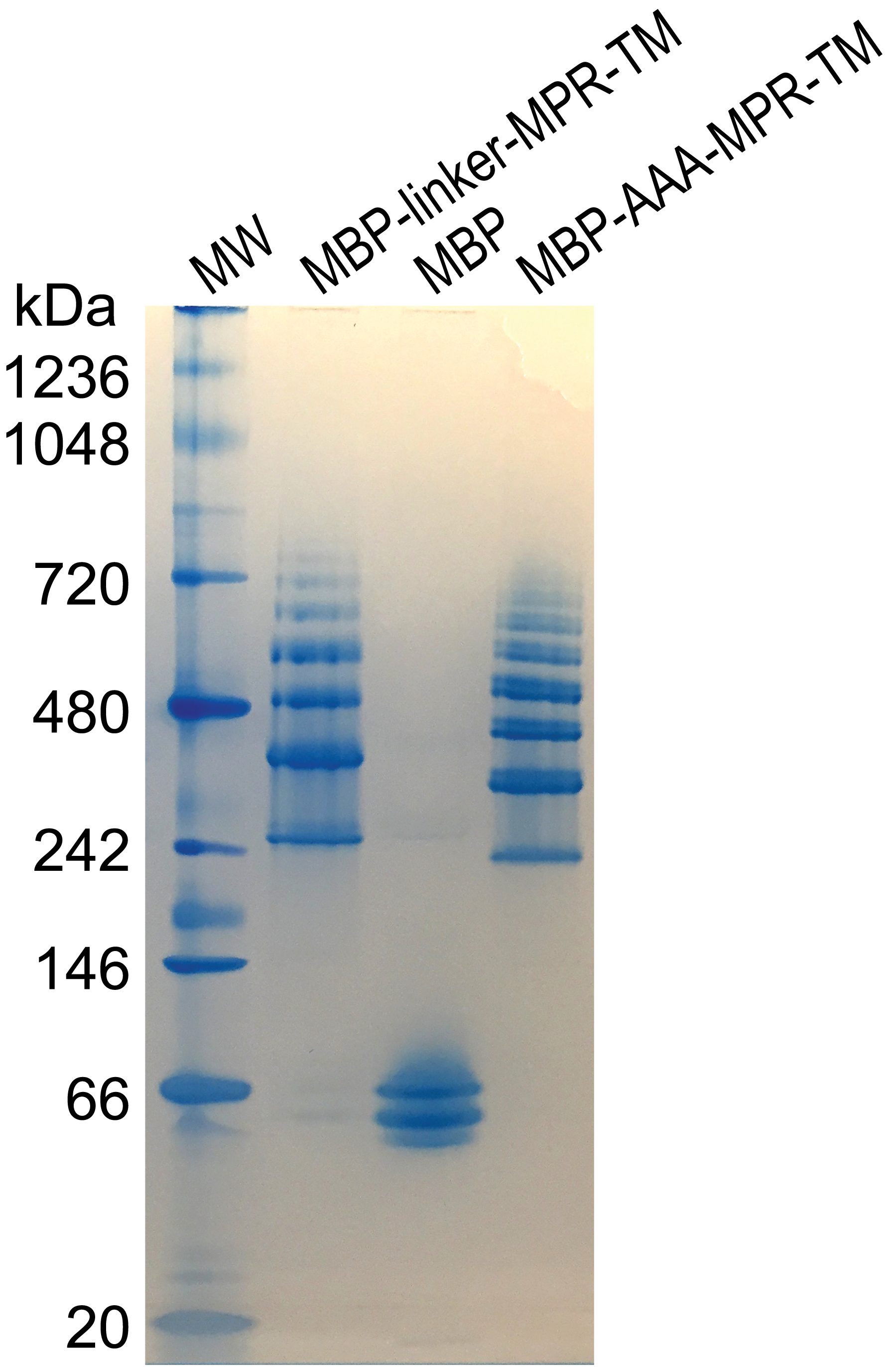

Supplement: S4 Fig — Native gels were prepared as described under Materials and Methods. Considering the very low polydispersity of our preparations seen by both SEC and DLS, it was expected that subjecting the protein to electrophoresis under non-denaturing conditions would result in a single band corresponding to the oligomeric protein. However, when preparations of the fusion protein were subjected to clear native PAGE (S4 Fig) as well as other nondenaturing PAGE protocols (data not shown) [41, 42] we observed a ladder pattern indicating multiple oligomeric forms. In contrast, the cleaved MBP fusion partner resolves as a monomer (S4 Fig). Pre-stained protein standards resolve according to their molecular masses, however the MBP-linker-MPR-TM protein migrate according to a more complex (largely empiric) function of its charge and mass. In addition, the electrophoresis was conducted in the absence of detergents (in either the gel or the running buffer), and the β-DDM present in the protein samples was expected to be progressively diluted during the run. Consequently, assessing the molecular mass of the protein bands and determining their oligomeric configuration is speculative and is probably a consequence of the PAGE. Two plausible interpretations are offered in S3 Table. (TIF) [file pone.0136507.s004.tif]

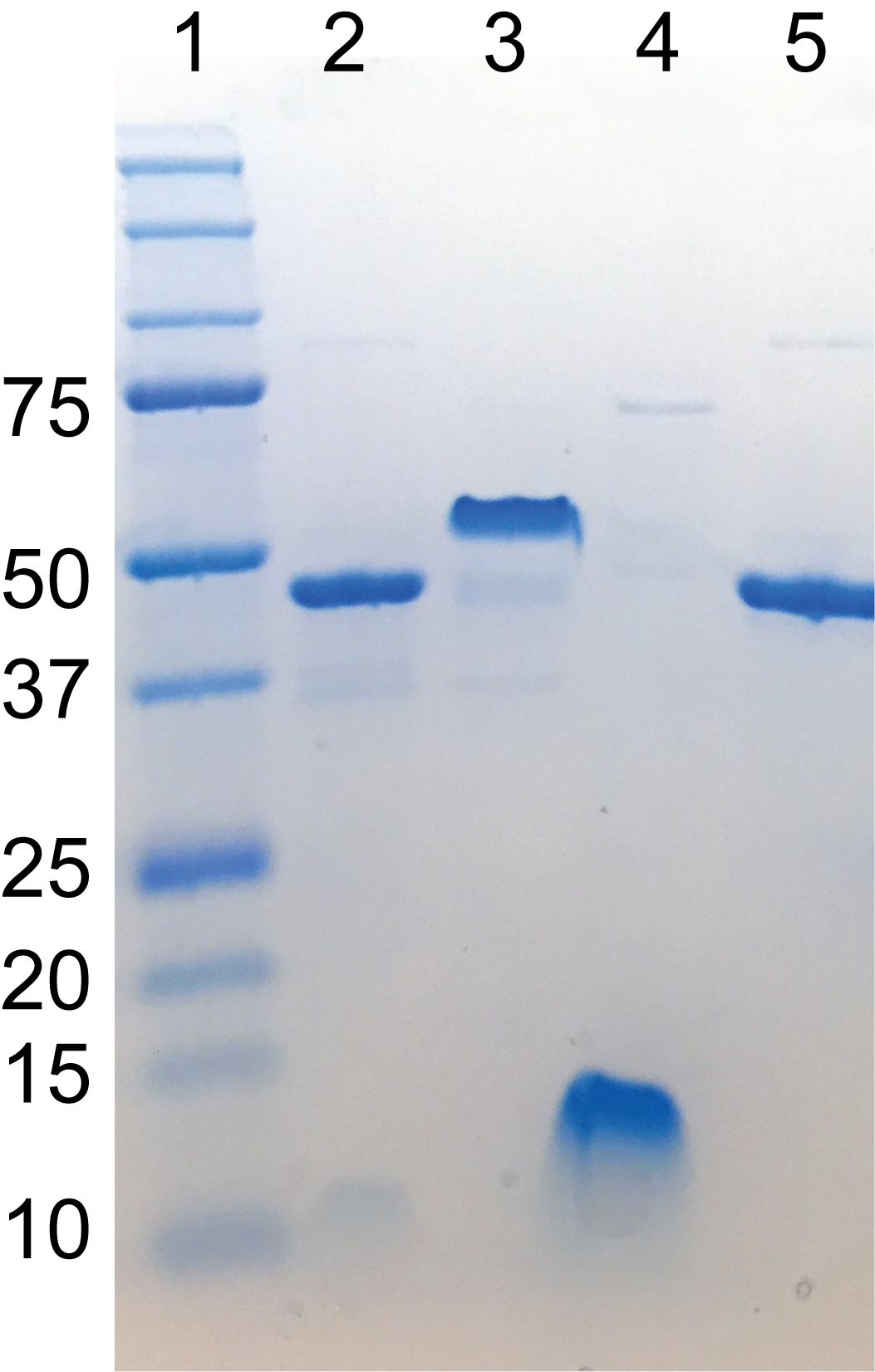

Supplement: S5 Fig — SDS-PAGE analysis of purification fractions stained by coomassie blue. Lane 1: proteins standards; lane 2: TEV cleavage products; lane 3: negative control (no TEV protease was added); lane 4: Ni-NTA flowthrough containing MPR-TM; lane 5: Ni-NTA elution containing MBP and TEV protease. (TIF) [file pone.0136507.s005.tif]
