# Supplementary material for: Biophysical Characterization of a Vaccine Candidate against HIV-1: The Transmembrane and Membrane Proximal Domains of HIV-1 gp41 as a Maltose Binding Protein Fusion
Source: PLoS One. 2015 Aug 21;10(8):e0136507. doi: 10.1371/journal.pone.0136507 (PMC4546420; doi:10.1371/journal.pone.0136507)
Supplement: S1 Table — (DOCX) [file pone.0136507.s006.docx]

## Supporting Information Tables

### Table S1. Primer sequences.

|  | |
| --- | --- |
| **Primer** | **Sequence^a^ (5’ to 3’)** |
| MBP-MPR-fuseF1 | CTGATTTATAACAAAGATCTGCTGCCGAACCCG |
| insert1-PCR1R | CAGGGCTgCATCGACAGTC |
| insert1-PCR2R | ATTAGTCTGCGCGgCtgcCAGGGCtgCATCGACAGTC |
| insert2-PCR1F | GCAGCTGCCATGGGATCTCAAACTCAACAAGAGAAG |
| MBP-MPR-fuseR1 | CGATGGTACCGTCGACGTCCTACAGGCGCGCC |
| MBP-MPR-fuseF2 | GCCGCGCAGACTAATgcagctgccATGGGATCTC |

^a^Restriction sites that were used are underlined. Nucleotide changes that differ from the original sequence are shown in lower case letters.

Mw-R: molecular weight estimated from the measured hydrodynamic radius of the analyte.
