# Supplementary material for: Biophysical Characterization of a Vaccine Candidate against HIV-1: The Transmembrane and Membrane Proximal Domains of HIV-1 gp41 as a Maltose Binding Protein Fusion
Source: PLoS One. 2015 Aug 21;10(8):e0136507. doi: 10.1371/journal.pone.0136507 (PMC4546420; doi:10.1371/journal.pone.0136507)
Supplement: S2 Table — (DOCX) [file pone.0136507.s007.docx]

## Supporting Information Tables

### Table S2. DLS measurement of purified MBP-linker-MPR-TM.

| **Intensity Distribution** | **Radius (nm)** | **Polydispersity (%)** | **Mw-R^a^ (kDa)** | **Intensity (%)** | **Mass (%)** |
| --- | --- | --- | --- | --- | --- |
| Peak 1 | 7.7 ± 0.5 | 13.4 | 397 | 100 | 100 |

^a^Mw-R: molecular weight estimated from the measured hydrodynamic radius of the analyte.

weight estimated from the measured hydrodynamic radius of the analyte.
