# Supplementary material for: Biophysical Characterization of a Vaccine Candidate against HIV-1: The Transmembrane and Membrane Proximal Domains of HIV-1 gp41 as a Maltose Binding Protein Fusion
Source: PLoS One. 2015 Aug 21;10(8):e0136507. doi: 10.1371/journal.pone.0136507 (PMC4546420; doi:10.1371/journal.pone.0136507)
Supplement: S3 Table — (DOCX) [file pone.0136507.s008.docx]

## Supporting Information Tables

### Table S3. MPR-TM fusion proteins resolve as regularly spaced bands by clear native PAGE

|  | | | | | | | | |  |  |
| --- | --- | --- | --- | --- | --- | --- | --- | --- | --- | --- |
|  | | Possible oligomeric state | Expected MW  (kDa) | | | | Bands’ Observed MW (kDa) | | 95% conf.? | |
|  | | Trimers  × *N* | protein | β-DDM | | total | Mean (95% confidence limits) | |  | |
| MBP-Linker-MPR-TM | | 1 | 156 | 120 | | 276 | 276 | (216-337) | OK | |
| Monomer MW = 53 kDa | | 2 | 312 | 60 | | 372 | 381 | (313-448) | OK | |
|  | | 3 | 468 | 30 | | 498 | 487 | (417-558) | OK | |
|  | | 4 | 624 | 15 | | 639 | 589 | (519-659) | OK | |
|  | | 5 | 780 | 8 | | 788 | 681 | (613-749) | Out | |
|  | | 6 | 936 | 4 | | 940 | 756 | (691-821) | Out | |
| MBP-AAA-MPR-TM | | 1 | 135 | 120 | | 255 | 253 | (194-312) | OK | |
| Monomer MW = 45 kDa | | 2 | 270 | 60 | | 330 | 347 | (282-412) | OK | |
|  | | 3 | 405 | 30 | | 435 | 432 | (363-502) | OK | |
|  | | 4 | 540 | 15 | | 555 | 517 | (446-587) | OK | |
|  | | 5 | 675 | 8 | | 683 | 581 | (511-651) | Out | |
|  | | 6 | 810 | 4 | | 814 | 646 | (577-715) | Out | |
|  | | D imers  × *N* | protein | β-DDM | | total | Mean (95% confidence limits) | |  | |
| MBP-Linker-MPR-TM | | 2 | 212 | 60 | 272 | | 276 | (216-337) | OK | |
| Monomer MW = 53 kDa | | 3 | 318 | 60 | 378 | | 381 | (313-448) | OK | |
|  | | 4 | 424 | 60 | 484 | | 487 | (417-558) | OK | |
|  | | 5 | 530 | 60 | 590 | | 589 | (519-659) | OK | |
|  | | 6 | 636 | 60 | 696 | | 681 | (613-749) | OK | |
|  | | 7 | 742 | 60 | 802 | | 756 | (691-821) | OK | |
| MBP-AAA-MPR-TM | | 2 | 196 | 60 | 256 | | 253 | (194-312) | OK | |
| Monomer MW = 45 kDa | | 3 | 286 | 60 | 346 | | 347 | (282-412) | OK | |
|  | | 4 | 376 | 60 | 436 | | 432 | (363-502) | OK | |
|  | | 5 | 466 | 60 | 526 | | 517 | (446-587) | OK | |
|  | | 6 | 556 | 60 | 616 | | 581 | (511-651) | OK | |
|  | | 7 | 646 | 60 | 706 | | 646 | (577-715) | OK | |

^a^Mw-R: molecular weight estimated from the measured hydrodynamic radius of the analyte.
