# Supplementary material for: Biophysical Characterization of a Vaccine Candidate against HIV-1: The Transmembrane and Membrane Proximal Domains of HIV-1 gp41 as a Maltose Binding Protein Fusion
Source: PLoS One. 2015 Aug 21;10(8):e0136507. doi: 10.1371/journal.pone.0136507 (PMC4546420; doi:10.1371/journal.pone.0136507)
Supplement: S4 Table — (DOCX) [file pone.0136507.s009.docx]

## Supporting Information Tables

### Table S4. DLS measurements of MBP-linker-MPR-TM (10 mg/mL) subjected to prolonged incubation at 4 °C.

| **Day^a^** | **Intensity**  **Distribution** | **Radius (nm)** | **Polydispersity (%)** | **Mw-R^b^ (kDa)** | **Intensity (%)** | **Mass (%)** |
| --- | --- | --- | --- | --- | --- | --- |
| 1 | Peak 1 | 7.5 ± 0.4 | 10.9 | 370 | 100.0 | 100.0 |
| 3 | Peak 1 | 7.6 ± 0.4 | 11.5 | 385 | 100.0 | 100.0 |
| 7 | Peak 1 | 7.1 ± 0.2 | 6.1 | 330 | 90.5 | 99.9 |
|  | Peak 3 | 90.2 ± 4.4 | 9.7 | 126571 | 9.5 | 0.1 |

^a^Samples were taken at the indicated time points.

^b^Mw-R: molecular weight estimated from the measured hydrodynamic radius of the analyte.
